# Funding Health Care for People Experiencing Homelessness: An Examination of Federally Qualified Health Centers’ Funding Streams and Homeless Patients Served (2014–2019)

**DOI:** 10.3390/ijerph21070853

**Published:** 2024-06-29

**Authors:** Marcus M. Lam, Nathan J. Grasse

**Affiliations:** 1 School of Leadership and Education Sciences, University of San Diego, San Diego, CA 92110, USA; 2 School of Public Policy and Administration, Carleton University, Ottawa, ON K1S 5B6, Canada; nathangrasse@cunet.carleton.ca

**Keywords:** Federally Qualified Health Centers (FQHCs), Health Care for the Homeless (HCH), government funding, people experiencing homelessness

## Abstract

It is estimated that three million people annually experience homelessness, with about a third of the homeless population being served by Federally Qualified Health Centers (FQHCs). Thus, FQHCs, dependent on government funding for financial viability, are vital to the infrastructure addressing the complex issues facing people experiencing homelessness. This study examines the relationship between various government funding streams and the number of homeless patients served by FQHCs. Data for this study come from three publicly available databases: the Uniform Data System (UDS), the IRS Core files, and the Area Resource File. Fixed-effects models employed examine changes across six years from 2014 to 2019. The results suggest that, on average, an additional homeless patient served increases the expenses of FQHCs more than other patients and that federal funding, specifically Health Care for the Homeless (HCH) funding, is a vital revenue source for FQHCs. We found that the number of homeless patients served is negatively associated with contemporaneous state and local funding but positively associated with substance use and anxiety disorders. Our findings have important implications for the effective management of FQHCs in the long term and for broader public policy supporting these vital elements of the social safety net.

## 1. Introduction

Homelessness is an intractable issue that requires interventions at multiple levels. At the macro level, public policies and elected officials are critical to driving government funding and public opinion to bring awareness to the homelessness problem and the subsequent consequences of inaction. At the micro level, interventions by social workers and medical professionals that help homeless persons find shelter and receive health care are critical to short-term survival and care. At the meso level, community-based not-for-profit organizations (NPOs) that employ medical professionals, social workers, and other frontline workers are an often overlooked but essential component of the multi-pronged solution to providing vital health, social, and shelter services to people experiencing homelessness.

While research has focused on the need for and/or effectiveness of macro- and micro-level interventions to address homelessness [1,2,3], less research has focused on meso-level interventions and the role that community-based NPOs play in not only direct service provision but also in empowering communities and promoting public health through the creation of social capital and community efficacy [4]. Indeed, urban sociologists note that community-based and not-for-profit organizations (NPOs) are vital to healthy and thriving communities. This is because organizations are not only service providers but also offer spaces for social interaction, informal resource exchange, formal community organizing activities, and leadership development [4,5]. These activities are vital for creating social capital, resilience, and a robust social safety net to promote personal and public health [4]. Thus, understanding solutions to address homelessness cannot be separated from understanding the community-based NPO organizations serving this population.

It is well documented that people experiencing homelessness have needs that extend beyond housing and shelter. These needs can include both basic and complex medical issues around mental health, substance abuse, trauma, or other behavioral issues [6,7]. An important group of organizations that provide crucial health services to the homeless population are Federally Qualified Health Centers (FQHCs). FQHCs are funded by the U.S. federal government, with some specifically designated to serve the homeless population. FQHCs are an integral component and comprise the largest subset of “safety-net providers” in the United States. Those participating in the Health Care for the Homeless (HCH) program are essential resources for people experiencing homelessness, as they provide accessible health care services and are located in underserved communities or areas of greatest need.

However, given their precarious financial circumstances leading to an inability to pay for services as well as unique healthcare needs, the cost of serving this population is often higher compared to patients who are not homeless. To provide services, FQHCs must rely on a number of revenue sources, including support from the government at all levels. Thus, understanding the costs and financial resources related to health care services for people experiencing homelessness is crucial for the effective management of FQHCs in the long term and for broader public policy in support of these vital safety-net organizations.

The following research questions guide this study: (1) How are services to populations experiencing homelessness associated with FQHC expenses? (2) To what extent do various types of revenue support FQHCs in serving populations experiencing homelessness? We draw from three publicly available databases to address these research questions: the Uniform Data System (UDS), the IRS Core files, and the Area Resource File. We model total expenses as well as the number of homeless patients served across six years, from 2014 to 2019, to explore the associations between major FQHC funding sources and the number of homeless patients served.

### 1.1. FQHC Revenue Structure and Service Outcomes

FQHCs are federally funded community health centers and have specific mandates to provide services to uninsured and underserved clients regardless of their ability to pay; to locate in underserved, rural, or high-need communities; to safeguard client voice in governance structures; and to provide enabling services such as transportation and language translation services to ensure accessibility and utilization to the most vulnerable and underserved clients [7,8].

To be effective, FQHCs must have adequate resources to operate their facilities and programs, and financial resources are the most basic type of resource. Indeed, an organization’s financial capacity is a critical precursor to organizational capacity [9]. Organizational capacity refers to staff quantity and expertise as well as to the organizational infrastructure and assets, both monetary and non-monetary (such as volunteers) required to carry out an organization’s mission effectively. For nonprofit organizations, the business model, or how nonprofits earn income, differs from traditional for-profit businesses in several ways. First, the clients who receive services from nonprofit organizations are often not the ones who pay for the services. In fact, nonprofits generally provide services to clients regardless of their ability to pay. Thus, nonprofits must engage in fundraising in the form of grant writing to philanthropic and government organizations, holding fundraising events, or soliciting donations from individuals to cover the full cost of their services. For example, FQHCs, on average, receive over a third of total revenue from grants [10]. Often, revenue from funders carries restrictions and only allows the recipient to spend the funds on specific activities. As such, nonprofits may be unable to use excess government or foundation grant funds to build reserves or savings.

For FQHCs, an important source of funding comes from the U.S. Human Resources and Services Administration’s (HRSA) Health Center Program [11]. In 2022, funding for Community Health Centers amounted to about USD 5.8 billion [12]. In addition to federal grants, FQHCs generate much of their income from Medicaid patients [8] through cost reimbursements (like hospitals and other health clinics that serve clients with private insurance). Medicaid is a public health insurance program for low-income individuals. To be eligible for Medicaid, an individual must fall into certain demographic categories (i.e., pregnant women, parents, children, seniors and people with disabilities, and in Medicaid expansion states, childless adults) and have income below a percentage of the federal poverty level (FPL) of USD 24,860 for a family of three and USD 14,580 for an individual (i.e., for childless adults, the threshold is 138% of the FPL) [13]. Approximately 94 million Americans are enrolled in the Medicaid program [13]. Clinics that serve Medicaid patients are reimbursed by both the U.S. federal government and each state based on specific rates set by the federal government, and states may also create a payment scheme that is equal to or greater than the federal payment scheme [14]. Medicaid insurance payments accounted for about 44% of total FQHC revenue in 2015, making Medicaid the largest revenue source for FQHCs [14].

However, both Medicaid reimbursements and grant funding may not cover the full cost of services (or may require a long wait time for reimbursement; thus, FQHCs must find alternative ways to cover this gap in cash flow) [15]. In addition, specific government grants may be restricted and may not be used for overhead or operational costs. Thus, FQHCs often have multiple funding streams to meet current expense obligations or to build reserves to address unexpected economic, social, or public health shocks that may disrupt their income sources or revenue streams. Thus, while revenue earned from Medicaid reimbursements is generally unrestricted, prior research has shown that FQHC operating margins are generally low [10]. In particular, FQHCs that serve specific populations, such as homeless or migrant populations, typically have lower margins and, thus, lower reserves than other FQHCs.

An organization’s funding structure inevitably influences the types of clients served, programmatic offerings, and, ultimately, service outcomes. For example, organizations that rely on fee-for-service income, such as student tuition in the case of higher education institutions or private insurance reimbursements in the case of hospitals, may be more likely to cater services to only fee pay clients to the detriment of indigent patients or focus on the most lucrative services (i.e., surgical procedures versus preventative medicine). In fact, not-for-profits with a high percentage of fee-for-service or earned income as opposed to donative or grant income are referred to as “commercial nonprofits” or “for-profits in disguise” [16]. Indeed, the relationship between financial strategy and capacity for client outcomes is well documented across a variety of nonprofit service fields. In arts and culture, earned income has been linked to performance measures such as greater attendance and attendance size [17,18]. Among environmental-based NGOs, researchers have found a link between resource tactics and increased mobilization efforts [19]. In the healthcare field, a review of the literature found studies reporting that grant funding is associated with increasing service sites, federal grants related to 24 h service and mental health treatment and counselling, federal and state grants associated with crisis support services, and grant funding with levels of uncompensated care [20].

### 1.2. FQHCs and Special Populations: Health Care for the Homeless (HCH) Program

The healthcare needs of the population experiencing homelessness are complex [6,7]. As such, estimates of the healthcare costs of serving this population range widely depending on illness severity and care setting (i.e., emergency room vs. clinic). Estimates range from about USD 25,000 for hospitalization to USD 1300 for an outpatient visit [21]. For the homeless population with associated substance disorders, the costs can range from USD 10,000 to USD 36,000 [22].

The Health Care for the Homeless (HCH) program was created in 1987 and supports FQHCs in providing specific services to people experiencing homelessness. Total funding from the U.S. Human Resources and Services Administration (HRSA) for the HCH program was approximately USD 503 million in 2022 [10]. Additional sources of support include funding from the federal Substance Abuse and Mental Health Service Administration (SAMHSA) (i.e., mental health and substance abuse block grants), the Department of Housing and Urban Development (HUD), and the federal Medicaid/Medicare programs [10,11]. Thus, overall funding for the HCH program includes a variety of direct and indirect grants and reimbursement schemes.

Services are offered through multiple modalities, including at specific health center locations, non-health-related community service sites such as soup kitchens and shelters, and mobile vans [23]. This allows services to be accessible to the most hard-to-reach homeless populations. The HCH program aims to address the multifaceted health needs of people experiencing homelessness. Thus, HCH grant recipients provide health screenings, emergency services, substance use services, dental care, behavioral case and general case management, among others [23]. HCH recipients provided services to approximately one million people experiencing homelessness in 2022, and health centers overall, regardless of whether they are HCH recipients, served approximately 1.3 million people experiencing homelessness [23,24].

## 2. Materials and Methods

### 2.1. Data Sources

The data sources for this study were drawn from three publicly available databases: the Uniform Data System (UDS) for organizational-level data on FQHC services (data source: https://data.hrsa.gov/data/download (accessed on 1 April 2021)); the IRS Core files for organizational-level data to measure FQHC financial capacity (data source: https://nccs-data.urban.org/data.php?ds=core (accessed on 1 May 2021)); and the Area Resource File for county-level data (data source: https://data.hrsa.gov/data/download) (accessed on 1 May 2021).

Federal legislation—Section 330 of the Public Health Service (PHS) Act—requires that FQHCs annually report financial, staffing, and patient demographic, service and utilization data [25]. These annual data are compiled in the UDS, offer consistent data on health centers across the U.S., and allow for comparisons between health centers and within individual health centers across time [25]. As such, the UDS is the most comprehensive dataset on FQHC outcomes and outputs available to researchers. For this study, data were analyzed from 2014 to 2019.

Data for total expenses and program revenue were drawn from the IRS Form 990 Core files. The Core files are datasets compiled from annual filings of IRS Form 990, the organizational tax returns for tax-exempt organizations. Data are available from 1989 to 2019. The universe of organizations in the Core files are all active and reporting registered 501c(3) charities.

The Area Health Resource File (AHRF) is a dataset compiled by the U.S. Bureau of Health Workforce and updated annually. It contains a range of health-related variables at the U.S. county level. Variables include data on the number of health professions, the number of health institutions such as hospitals and health centers, as well as the health and socio-economic characteristics of the population such as poverty level, age, gender identification, or health insurance status.

### 2.2. Variables

#### 2.2.1. Outcome, Models 1–3, Total Expenses

In order to measure the FQHCs’ expenses, we use data on their total reported expenses from the IRS Form 990 (Part 1, Line 18).

#### 2.2.2. Outcome, Models 4–6, Service to Patients Experiencing Homelessness

Our primary outcome is the number of patients served per year who have experienced homelessness (UDS Table 4, line 23) [25]. Specifically, “Total Patients Experiencing Homelessness” (line 23) is where respondents report “the total number of patients known to have experienced homelessness at the time of any service provided during the calendar year, even if their housing situation changed during the year”. (UDS Manual, 2022, p. 42) [25]. The UDS Manual provides instructions for reporting on homelessness:

“Report patients who lack housing. Include patients whose primary residence during the night is a supervised public or private facility that provides temporary living accommodations. Include patients who reside in transitional housing or permanent supportive housing”.

This total (line 23) is comprised of the following categories (see Table 1 for a summary of all variables) [25]:

- Patients living in a temporary shelter for individuals experience experiencing homelessness (line 17).
- Patients living in temporary transitional housing, excluding those “transitioning from jail or those residing in or transitioning from an institutional treatment program, the military, schools, or other institutions”. (UDS Manual, 2022, p. 43) (line 18).
- Patients temporarily living with others (“doubled up”), excluding co-tenant rentals (line 19).
- Patients living on the street, including individuals “living outdoors, in a vehicle, in an encampment, in makeshift housing/shelter, or in other places generally not deemed safe or fit for human occupancy”. (p. 43) (line 20).
- Patients who are living in permanent supportive housing or who were housed when first seen but have since experienced homelessness during the previous 12 months (lines 21 and 21a).
- Other circumstances in which patients are known to be experiencing homelessness but housing arrangements are unknown (line 22).

#### 2.2.3. Independent Variables

We examine three categories of independent variables: service population variables, revenue variables, and county-level variables to assess associations between characteristics in the service populations of FQHCs, the revenues they generate, location characteristics, and the number of homeless patients served.

##### Service Population Variables

Given the associations between homelessness and mental health and other substance use disorders at the individual level, we wanted to account for whether these factors were present in FQHCs’ service populations. While we cannot speak to individual-level correlations, their combined presence in organizations’ service populations would likely pose challenges for FQHCs. We include the total number of patients seen with alcohol-related disorders (UDS Table 6a, line 18, column b), other substance use disorders excluding tobacco (UDS Table 5a, line 19, column b), and anxiety disorders (UDS Table 6a, line 20b, column b).

##### Revenue Variables

We also include variables to measure the revenue and support FQHCs rely on to fund their operations. This includes community health center funding (Table 9E, line 1b Ca). We also include the amount of “Health Care for the Homeless” (HCH) grants reported in the UDS in Table 9E, line 1c, as we expect this revenue will be critical for homeless serving FQHCs. We also include funding from public housing primary care (T9E, line 1e), U.S. Bureau of Primary Health Care (BPHC) grants (T9E, line 1), other federal grants (T9E, line 5), state government grants and contracts (T9E, line 6), local government grants and contracts (T9E, line 7), foundation private grants and contracts (T9E, line 8), as well as specific state and local funding for indigent care programs (T9E, line 6a), and total other non-patient-related revenue (Table 9E, line 10). To understand whether organizations’ programs generate support that might fund services to people experiencing homelessness, we also include funding from fees or earned income (i.e., program service revenue from Form 990 Part 1, Line 9). Including these data from FQHCs’ tax filings is useful, as program service revenues are frequently redacted from publicly available UDS data (Table 9). All revenue variables were inflation-adjusted.

##### County-Level Variables

Finally, to consider the effects of the service and demand environment that may be associated with the number of people experiencing homelessness, we include five covariates at the county level. First, we include an estimate of the population to control for size. We also include the number of community mental health centers as a control for the other resources available in a community to serve the homeless population. To control for covariates that may drive homelessness, we consider the percentage of persons in poverty, the percentage of those under 65 years old without health insurance, and a dichotomous variable that measures whether a county is designated as a shortage area for mental health professionals (1 = entire county is a shortage area; 2 = part of a county is a mental health underserved area) [26,27].

### 2.3. Cleaning Procedure

The original data file contained observations for five reporting years from 2015 to 2019. We excluded organizations in U.S. territories such as Guam, the Virgin Islands, American Samoa, etc. The number of reporting organizations per year ranged from 1331 to 1352. Of these organizations, 1306 reported data for all five years, ten organizations for four years, 25 for three years, 12 for two years, and 38 for only one year.

Next, we created a “match key” to link the UDS data with the IRS Core dataset. To create the “match key”, a research assistant looked up the employer identification number (EIN) for each organization in the UDS dataset. Using the match key, we merged the IRS Core file with the UDS dataset using the EIN and a unique FQHC identification number.

Organizational data on homeless patients served were also sometimes removed from the UDS database. We expect that these data were purposefully redacted, primarily due to the low numbers of homeless patients in their service populations; we did not want to impute zeros for these organizations, so organizations were excluded if they did not report homeless patient data in at least five of the six years from 2014 to 2019; this led to the exclusion of 1046 observations in our the panel of 6428 organization-years (16.27% of the observations).

### 2.4. Analysis Procedure

We rely on fixed-effects panel regression models to identify the associations between the independent and dependent variables. These account for fixed effects by city, state, year, and organization using the Stata package “reghdfe” and its associated commands. These multi-fixed-effects models identify the residuals of each modelled variable given the fixed effects and then regress these [28] on the dependent variable; the results are equivalent to utilizing dummy variables for each fixed effect but substantially more efficient when the number of fixed effects is large, as in this case [28] (package available at https://github.com/sergiocorreia/reghdfe (accessed on 1 March 2022).

## 3. Results

### 3.1. Descriptive Results

**Figure 1:**
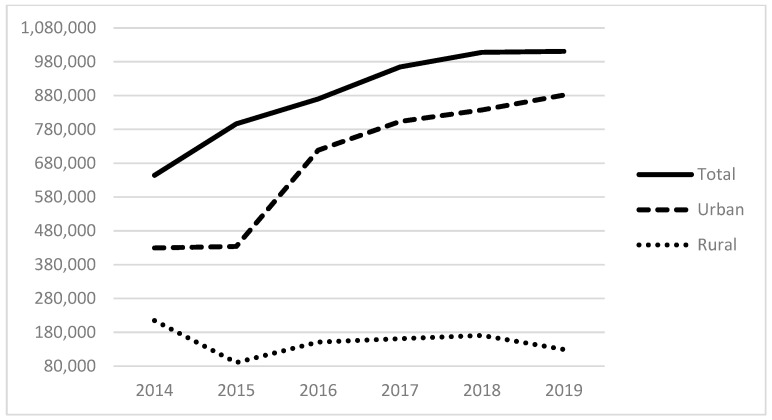
The total homeless patient service population from reporting FQHCs.

**Figure 2:**
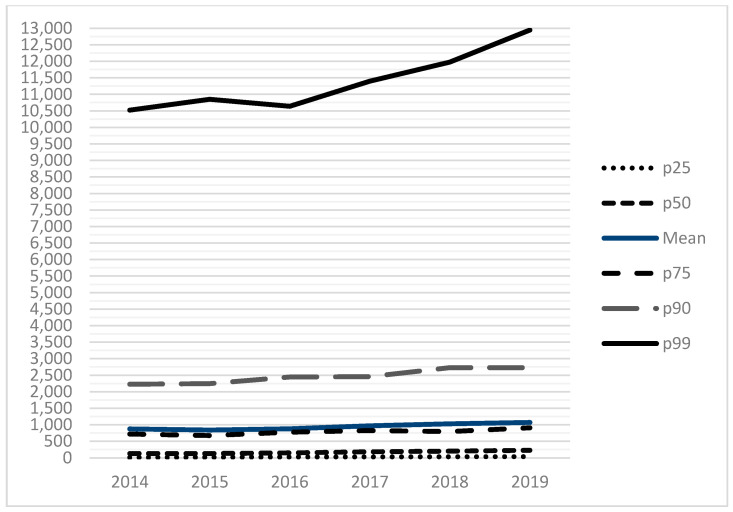
The total homeless patient service population by percentile.

Table 1 (below) presents a descriptive summary of all variables for 2019. The average number of homeless patients served was 1125, with a median of 239. This suggests the importance of a few large providers for the provision of care, evidenced also by looking at the 75th and 90th percentiles. Figure 1 and Figure 2 below display the total number of patients served by all FQHCs from 2014 to 2019 (Figure 1) and the percentile distribution of these homeless service populations (Figure 2). These demonstrate the growth in the total size of the FQHCs’ reported collective homeless service population, from 644 K in 2014 to over 1 M in 2019 (Figure 1). Figure 2 demonstrates that the vast majority of this service occurs in the top quartile of FQHCS. For example, in 2019, an organization in the 99th percentile served nearly 13,000 homeless patients compared to nearly 3000 for the 75th percentile and about 1000 for the 50th or median percentile. This demonstrates that the group of organizations in the top quartile (75th percentile and above) serve the vast majority of homeless patients and are essential to these services. We also examine the total expenses of FQHCs, finding the mean total expenses were USD 30.6 M in 2019. At the county level, the sample is distributed across 545 counties with an average of about 357,000 people per county and a median of about 117,900 people; on average, 14% of the population in a county are in poverty, and 11% who are under 65 have no health insurance; there are typically few community mental health centers within our sample counties, with an average of less than one (0.14), a median of zero, and a maximum of 14. The majority of the counties are full or partial mental health shortage areas.

**Figure 3:**
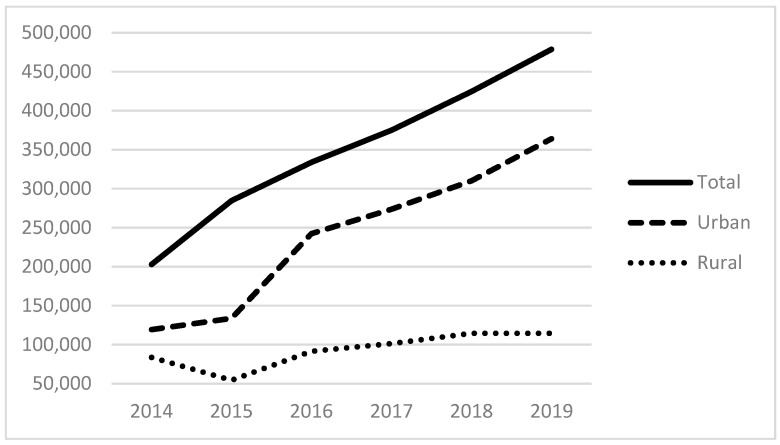
Patients with non-alcohol and non-tobacco substance abuse disorders in the service populations of reporting FQHCs.

**Figure 4:**
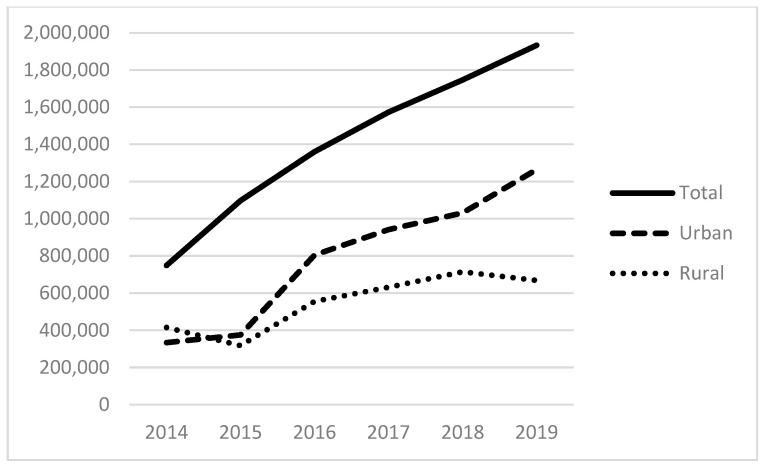
Patients with anxiety disorders in the service populations of reporting FQHCs.

Examining our independent variables, the average number of patients served that experience alcohol-related disorders is 311; those with other substance-related disorders are 525; and those with anxiety disorders are 2083, with each of these also positively skewed. Figure 3 and Figure 4 provide the total number of patients served for substance use (non-alcohol and non-tobacco) and anxiety disorders. The total number of patients with substance use disorders has more than doubled since 2014, totaling more than 450,000 patients in 2019 (Figure 3). The total number of patients seen with anxiety disorders also more than doubled between 2014 and 2019 to almost 2 million patients. As with the size of the homeless population above and the revenues below, there are distinct differences between urban and rural FQHCs.

With regard to government funding, the most FQHC support comes from BPHC grants, with an average grant of USD 3.9 million. BPHC funding is followed by Community Health Center grants, which average about USD 3.2 million. State and local grants and contracts account for the next largest, contributing an average amount of about USD 651,000. Funding by state and local indigent care programs provides an average grant size of about USD 503,000. The HCH program provided average grants of about USD 300,000. Beyond government sources, funding from private foundation grants and contracts is about USD 708,000 on average, and other revenue sources account for, on average, USD 983,000. Finally, the largest funding source, by far, comes from program revenue, including Medicaid reimbursements, with an average amount of USD 26 million.

**Figure 5:**
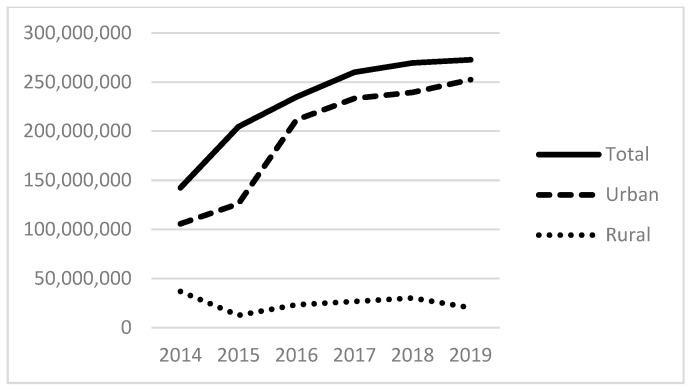
Total dollars of health care for the homeless (HCH) funding to FQHCs.

**Figure 6:**
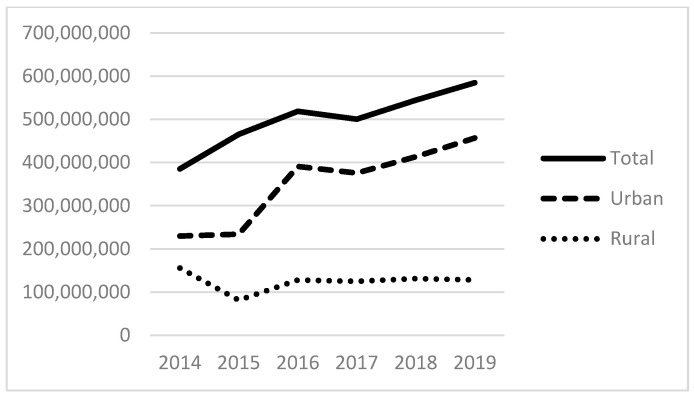
Total dollars of state grants and contracts to FQHCs.

**Figure 7:**
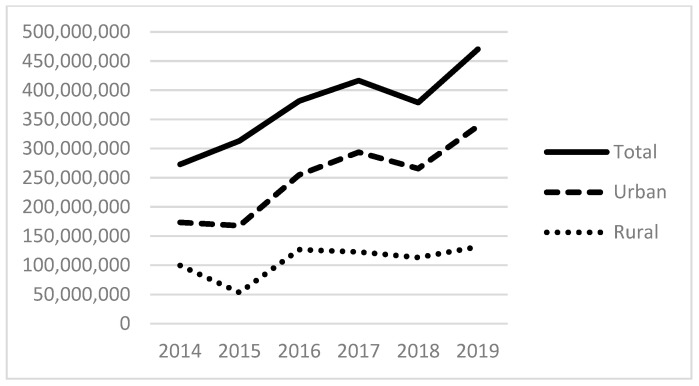
Total dollars of state and local indigent care funding to FQHCs.

Figure 5, Figure 6 and Figure 7 show the total amount of funding by funding source for the years under study (2014–2019). The HCH program provided nearly USD 300 million in 2019, with the majority of funds going to programs located in urban areas. Total state and local grants and contracts to FQHCs totaled USD 600 million in 2019. State and local funding specifically for indigent care totaled more than USD 400 million in 2019.

At the county level, the average-sized county had a population of 1.12 million. In terms of need, persons in poverty average about 14% by county and on average, about 11% of people under 65 have no health insurance. Virtually all FQHCs are located in counties where at least part of the county is designated as a health professional shortage area in terms of mental health providers. The number of health centers also reflects this; at least half of FQHCs are in counties with zero mental health centers.

### 3.2. Regression Model Results

Our first models, in Table 2 below, examine the total expenses of FQHCs, predicting these with the number of homeless patients, the total number of other patients, and the county-level variables mentioned above. These demonstrate that additional expenses are associated with increases in the number of homeless served and increases in any other type of patients when examining all FQHCs (Model 1) and urban FQHCs (Model 2), but the expense of serving homeless patients is likely greater. While not the primary focus of our analysis, these models demonstrate why the revenue structures of homeless-serving FQHCs are essential. They will need to cover the expenses associated with the presence of homeless patients in their service populations. In our models of all FQHCs (1) and urban organizations (2), serving additional homeless patients is predicted to increase expenses by almost twice as much as additional non-homeless patients.

Results examining the total number of homeless patients served, found in Table 3 below, are presented in three models: (4) including all FQHCs, (5) examining urban FQHCs, and (6) examining rural FQHCs. We focus this discussion on models (4) and (5), but we are hesitant to interpret (6) as the F value for this model (0.287) indicates that the independent variables are not jointly significant predictors of the number of homeless patients served in rural FQHCs. Examining model (4), we see that both the presence of other substance abuse disorders and anxiety disorders in the patient population are associated with larger populations of homeless service recipients. These are aggregate counts by FQHC rather than individually measured variables, so we cannot speak to the overlap of these characteristics at the individual level. Still, they reflect the likely prevalence of other disorders in the service populations of homeless-serving FQHCs, with associations of about 35% and 23% for patients with other substance abuse disorders and patients with anxiety disorders and the number of homeless patients served, respectively. The other takeaway from model (4) is the association between Health Care for the Homeless funding and the provision of service to homeless patients in FQHCs, which are more than double the size of the coefficients of any other revenue source. While this is not a surprising result, it does reflect the likely importance of this revenue stream for FQHCs, with about USD 825 of Health Care for the Homeless funding associated with one homeless patient served, holding other factors constant (0.00121 × 825 = 0.998).

Looking at the results of model (5), we see similar patterns among urban FQHCs, with positive associations between substance abuse disorders and anxiety disorders in service populations. We also see a negative association between the number of alcohol-related disorders in FQHCs’ service populations and the number of homeless patients in their service populations. While these cannot be interpreted as anything other than associations, they highlight each characteristic’s relative prevalence in homeless-serving urban FQHCs (as seen for other substance abuse disorders in Figure 3 and anxiety disorders in Figure 4).

Examining the revenue variables, the critical nature of Health Care for the Homeless funding to homeless-serving urban FQHCs becomes clear. Holding other variables constant and accounting for multiple fixed effects, about USD 840 of funding (0.00119 × 840 = 0.9996) is associated with an additional homeless service recipient in these organizations, but other revenues are negatively associated with the number of homeless patients served. Bureau of Primary Health Care (BPHC) grants, state and local indigent care program funds, and local government grants and contracts are associated with fewer homeless patients served. Of course, we do not mean to suggest a causal relationship between increases in these funds and reduced numbers of homeless service recipients. Still, negative associations indicate that these revenues are less available to urban FQHCs when serving homeless patients. We note that urban FQHCs served most of the patient population from 2014 to 2019 (Figure 1). The county-level covariates are included to ensure that local factors are not responsible for patterns of service provision. None of these are statistically significant.

## 4. Discussion

The National Health Care for the Homeless Council (NHCHC) estimates that there may be up to three million people a year who experience homelessness [29]. In addition, homelessness is related to a host of health conditions, while health problems can also lead to homelessness [29]. FQHCs serve about one million homeless patients; this accounts for roughly one-third of all people experiencing homelessness but also serve many other patients in their communities. As such, FQHCs not only offer vital health services to the homeless population but may also be seen as a preventative measure, providing health services for vulnerable populations, such as those who do not have health insurance or might not seek treatment for other reasons.

This exploratory analysis may offer new insights regarding health factors that are likely to be prevalent in the service populations of FQHCs serving homeless individuals. Specifically, current research suggests that homelessness and alcohol disorders are correlated at the individual level [30]. Our findings suggest a negative association between the number of patients who experience homelessness and patients who have alcohol-related disorders at the level of FQHC service populations. Instead, other substance use, as well as anxiety disorders, are positively correlated to the number of homeless served among FQHCs. While our findings are at the aggregate organization level, and we cannot generalize to individuals, they are consistent with those from others who find anxiety and substance use are important comorbid conditions with homelessness at the individual level [31]. However, FQHCs are estimated to serve only about one-third of the population experiencing homelessness, and it is likely that the association between alcohol use disorder and homelessness remains quite high, and individuals with alcohol use disorders are treated in other care settings, such as emergency rooms [32]. Additionally, prior research suggests that patients with risky alcohol behaviors are more likely to underutilize primary healthcare services; therefore, substantiated cases of alcohol-related disorders may be systematically undercounted [33]. For all these reasons, we interpret our finding of a negative association between alcohol-related disorders and the number of patients who experience homelessness with caution.

Overall, however, our findings suggest the complex physical and mental health issues likely to be present within urban homeless-serving FQHCs’ service populations and that organizations addressing homelessness are likely to require programs to address a broad spectrum of disorders, including other types of substance abuse and mental health disorders, likely increasing the difficulty and cost of providing services.

Given that our data are at the organizational level, we cannot provide evidence that individuals who experience homelessness also have anxiety and other substance use disorders; we only speak to their presence in the same service populations. We also cannot speak to patterns in the service populations of other types of homeless-serving organizations. We only suggest that the likely presence of these factors in the service populations of FQHCs treating homeless patients must create challenges and program needs for FQHCs located in urban areas, and we speculate this may raise the cost of service provision in these organizations. We believe this is worthy of future research.

Regarding revenue streams, this analysis evaluates multiple sources of funding, finding that contemporaneous local government funding is negatively associated with the number of homeless patients served. It also highlights the significance of the Health Care for the Homeless (HCH) program, which correlates with the number of homeless patients served. Indeed, findings from this study are consistent with the argument that organizations’ financial strategies are likely to be associated with programmatic outcomes such as the types of clients served. For example, in the healthcare field, grant funding is associated with community support services like uncompensated care, mental health treatment and counseling, or 24-h service [20], while fee-for-service funding may be related to serving higher-income clients and providing less preventive care. We cannot make any causal claims, nor do we suggest that specific funding streams (i.e., state and local funding) disincentivize people experiencing homelessness from certain services, only that the negative association between specific funding streams, such as state and local funding, may be related to the timing and receipt of payments.

A common experience by FQHCs receiving government grants may reflect a gap between the full cost of service and the funding provided by the grant. That is, the full cost of service, which includes indirect and direct expenses, is generally greater than the grant revenue [15,34]; thus, service providers must supplement grant revenue with other forms of income, such as fundraising dollars. Another common source of revenue is the fee for service in the form of cost reimbursements from government insurance programs like Medicaid and Medicare. However, given the structure of Medicaid funding, there is often a lag between when services are provided and when service providers receive reimbursements. That is, NPO service providers must absorb the upfront expenses of providing services and wait for funders to reimburse these costs. This wait time can range from a few weeks to a few months [35]. Thus, given the panel structure of our data, the negative association may simply result from the average lag time between service provision and grant receipt. This is supported by a positive association between the future value of state and local indigent care (T + 1) and the number of homeless patients (model not presented). In addition, grant amounts do not typically cover the full cost of services. Thus, nonprofits must make up the difference in costs through other means such as fundraising, as seen in high amounts of revenue from other sources (Table 1) or serving other client populations. These findings, taken together, point to broader challenges in managing multiple sources of income and working with government funders. They also demonstrate the importance of financial capacity and proper financial management to cope with the intricacies and unintended consequences (to the long-term financial sustainability of the organization and impacts on clients served) of multiple revenue streams.

Finally, the crucial role of federal funding in addressing homelessness cannot be over-emphasized. It is estimated that the cost of chronic homelessness to taxpayers averages about USD 35,000 per person per year (in 2017) [36]. Our results suggest that Health Care for the Homeless funding is likely a critical element of urban FQHCs’ revenue structures and that HCH funding makes an important contribution to serving homeless members of the community. It is crucial, therefore, to continue to fund FQHCs as a vital resource for people experiencing homelessness and as a cost-effective and preventative solution to the homelessness crisis. To the best of our knowledge, this is the first study to examine the associations between multiple revenue streams and the provision of services to people experiencing homelessness, specifically with regard to state and local funding. However, our findings suggesting the critical nature of federal funding and patient service capacity are consistent with prior studies. For example, Han et al. (2017) found that federal grant funding is positively related to medical and preventive service visits as well as the number of patients served [37]. Similarly, Jiao et al. (2022) found that increases in federal grants (specifically Section 330 grants) are associated with double the number of patient visits compared to Medicaid revenue [34].

## 5. Conclusions

Homelessness is a persistent and pressing issue that requires, ideally, a coordinated response by government agencies, policymakers, community-based organizations (including not-for-profit (NPO) community health centers), and an army of frontline service providers and volunteers. Indeed, FQHCs, which provide health services to individuals experiencing homelessness, are likely to be a vital component of any coordinated policy response. The primary findings from this study—that homeless patients are predicted to increase expenses by almost twice as much as additional non-homeless patients, and Health Care for the Homeless (HCH) funding is more than double the size of any other revenue source—offer insights into the specific funding streams likely to be essential to increasing the number of homeless patients served by FQHCs. Methodologically, this exploratory study demonstrates how organizational-level data can provide an alternative perspective on the scale and scope of homelessness, given the difficulty of counting the number of people experiencing homelessness. Future studies can examine the interaction of revenue streams with the number of homeless people served or the interaction of conditions related to homelessness. Additionally, they can explore specific enabling services for people experiencing homelessness, like mobile service sites or telemedicine, and outcomes related to preventative behavior among people without housing, such as immunizations or health screenings. Certainly, this study demonstrates the use of organizational-level data to explore important questions about homelessness from a meso perspective.

## Figures and Tables

**Table 1:** Descriptive statistics for all variables (reporting year 2019 only).

	N	p10	p25	p50	Mean	p75	p90	SD
Service Population Variables								
Homeless Patients	857	6	40	239	1125	946	2939	2565
Alcohol-related Disorders	857	39	79	179	311	355	709	453
Other Substance-related Disorders	857	43	111	257	525	608	1290	780
Anxiety Disorders	857	304	656	1343	2083	2479	4591	2489
Expenses (per $10K)								
Total Expenses	860	524	916	1820	3060	3420	6960	3790
Revenue Variables (per $10K)								
Community Health Center Funding	857	106	169	267	318	412	604	219
Health Care for the Homeless Fundi	857	0	0	0	30	0	105	82
Public Housing Primary Care Fundin	857	0	0	0	7	0	0	31
Total BPHC Grants	857	137	204	315	391	481	742	283
Total Other Federal Grants	857	0	0	3	36	33	103	85
State Government Grants and Contr	857	0	0	15	65	67	177	154
State and Local Indigent Care Progra	857	0	0	0	50	10	105	235
Local Government Grants and Contr	857	0	0	0	48	21	98	206
Foundation Private Grants and Cont	857	0	4	24	71	77	176	138
Other Revenue (non-patient)	857	0	4	15	98	52	151	846
Program Service Revenue T-1	857	191	445	1070	2600	2240	4650	15,100
County-Level Variables								
Population Estimate	545	21,039	39,371	117,873	356,911	379,468	873,972	774,431
Percent Persons in Poverty	545	8.63	10.87	13.64	14.34	17.17	20.68	4.77
Community Mental Health Centers	545	0.00	0.00	0.00	0.14	0.00	0.35	0.54
Percent Under 65 without Health Ins	545	6.10	7.80	10.10	11.12	13.82	17.43	4.60
Mental Health Underserved Area	545	1.00	1.00	2.00	1.61	2.00	2.00	0.51

**Table 2:** Total expenses of FQHCS.

	All FQHCs	Urban FQHCs	Rural FQHCs
	(1)	(2)	(3)
	Total Expenses	Total Expenses	Total Expenses
	b/(se)	b/(se)	b/(se)
Patient Population Variables			
Homeless Patients (T-1)	1604.9 ***	1461.2 ***	1155.8
	(348.5)	(317.2)	(980.1)
Total Patients Minus Homeless Patients (T-1)	848.3 ***	857.5 ***	765.2 ***
	(103.4)	(130.0)	(196.5)
County-Level Variables			
Population Estimate	5.547	1.480	203.1
	(7.911)	(7.379)	(180.8)
Percent Persons in Poverty	−410,972.4 ***	−386,551.8 *	−178,943.8
	(110,020.1)	(149,666.7)	(125,827.9)
CHCs in County	−616,062.2	−553,404.5	−2,460,629.0
	(397,418.7)	(395,453.4)	(2,656,254.2)
Percent Under 65 without Health Insurance	−494,419.9 *	−444,841.5	−482,886.5
	(216,519.1)	(273,435.5)	(324,153.4)
Mental Health Underserved Area	−1,205,313.4	−539,216.6	−872,567.8
	(772,957.5)	(890,049.7)	(863,647.7)
N	4201	2350	1698
Groups	917	532	393
Within R^2^	0.2206	0.2394	0.1644
Prob > F=	0.000	0.000	0.000

* *p* < 0.10; ** *p* < 0.05; *** *p* < 0.01.

**Table 3:** Models of the reported number of homeless patients treated by FQHCs.

	All FQHCs	Urban FQHCs	Rural FQHCs
	(4)	(5)	(6)
	Homeless Patients	Homeless Patients	Homeless Patients
	b/(se)	b/(se)	b/(se)
Service Population Variables (number of patients)			
Alcohol-related Disorders	−0.344	−0.441 **	−0.112
	(−1.52)	(−2.03)	(−0.53)
Other Substance-related Disorders (non-tobacco)	0.356 **	0.624 ***	−0.0218
	(2.32)	(2.72)	(−0.18)
Anxiety Disorders	0.232 ***	0.327 ***	0.0195
	(3.14)	(4.23)	(0.75)
Revenue Variables (per USD 10 K ADJ 2022)			
Community Health Center Funding	0.305	1.571	−1.129
	(0.27)	(1.22)	(−1.29)
Health Care for the Homeless Funding	12.01 ***	11.90 ***	2.207
	(3.19)	(3.12)	(0.75)
Public Housing Primary Care Funding	4.803	5.423	−9.369
	(1.46)	(1.47)	(−0.82)
Total BPHC Grants	−1.271	−1.943 **	1.143
	(−1.28)	(−2.21)	(1.56)
Total Other Federal Grants	−0.00880	0.437	−0.0924
	(−0.01)	(0.27)	(−0.40)
State Government Grants and Contracts	1.184	0.859	0.101
	(1.26)	(1.11)	(0.27)
State and Local Indigent Care Programs	−0.340	−1.844 *	0.0442
	(−0.72)	(−1.76)	(1.35)
Local Government Grants and Contracts	−0.456	−0.522 **	0.0588
	(−1.42)	(−2.07)	(0.03)
Foundation Private Grants and Contracts	−0.0740	−0.0156	−0.960 **
	(−0.50)	(−0.13)	(−2.17)
Other Revenue	0.235	0.246	−0.106
	(0.88)	(0.86)	(−0.60)
Program Service Revenue T-1	0.0932	0.128	0.0160
	(1.43)	(1.31)	(0.89)
County-Level Variables			
Population Estimate	0.000737	0.000818	−0.00838
	(0.65)	(0.67)	(−0.53)
Percent Persons in Poverty	−5.795	6.532	−15.14 **
	(−0.43)	(0.23)	(−2.07)
CHCs in County	−17.35	−42.01	150.4
	(−0.43)	(−0.96)	(1.37)
Percent Under 65 without Health Insurance	−6.202	−28.86	7.932
	(−0.34)	(−0.56)	(1.33)
Mental Health Underserved Area	−10.06	−33.19	22.71
	(−0.08)	(−0.15)	(0.33)
N	2936	1628	1160
Groups	896	512	368
Within R^2^	0.2536	0.3202	0.0559
Prob > F=	0.000	0.000	0.287

* *p* < 0.10; ** *p* < 0.05; *** *p* < 0.01.

## Data Availability

The original data presented in the study are openly available from (1) Uniform Data System (UDS) (https://data.hrsa.gov/data/download (accessed on 1 April 2021)); (2) IRS Core files (https://nccs-data.urban.org/data.php?ds=core (accessed on 1 May 2021)); and (3) Area Resource File (https://data.hrsa.gov/data/download (accessed on 1 May 2021)).
